# Protective Effects against *Brucella abortus* 544 Infection in a Murine Macrophage Cell Line and in a Mouse Model via Treatment with Sirtuin 1 Activators Resveratrol, Piceatannol and Ginsenoside Rg3

**DOI:** 10.4014/jmb.2209.09028

**Published:** 2023-01-17

**Authors:** Alisha Wehdnesday Bernardo Reyes, Heejin Kim, Tran Xuan Ngoc Huy, Trang Thi Nguyen, Wongi Min, Hu Jang Lee, Jin Hur, John Hwa Lee, Suk Kim

**Affiliations:** 1 Department of Veterinary Paraclinical Sciences, College of Veterinary Medicine, University of the Philippines Los Baños, College, Laguna 4031, Philippines; 2 Institute of Animal Medicine, College of Veterinary Medicine, Gyeongsang National University, Jinju 52828, Republic of Korea; 3 College of Veterinary Medicine, Jeonbuk National University, Iksan 54596, Republic of Korea

**Keywords:** *Brucella abortus*, piceatannol, ginsenoside Rg3, resveratrol, sirtuin 1

## Abstract

Brucellosis is a contagious zoonotic disease that infects millions of people annually with hundreds of millions more being exposed. It is caused by *Brucella*, a highly infectious bacterial species capable of infecting humans with an estimated dose of 10-100 organisms. Sirtuin 1 (SIRT1) has been reported to contribute to prevention of viral diseases as well as a chronic infection caused by *Mycobacterium bovis*. Here, we investigated the role of SIRT1 in the establishment of *Brucella abortus* infection in both in vitro and in vivo systems using the reported SIRT1 activators resveratrol (RES), piceatannol (PIC), and ginsenoside Rg3 (Rg3). In RAW264.7 cells, SIRT1 activators did not alter the adherence of *Brucella* or *Salmonella* Typhimurium. However, reduced uptake of *Brucella* was observed in cells treated with PIC and Rg3, and survival of *Brucella* within the cells was only observed to decrease in cells that were treated with Rg3, while PIC treatment reduced the intracellular survival of *Salmonella*. SIRT1 treatment in mice via oral route resulted in augmented *Brucella* resistance for PIC and Rg3, but not RES. PIC treatment favors Th2 immune response despite reduced serum proinflammatory cytokine production, while Rg3-treated mice displayed high IL-12 and IFN-γ serum production. Overall, our findings encourage further investigation into the complete mechanisms of action of the different SIRT1 activators used as well as their potential benefit as an effective alternative approach against intracellular and extracellular pathogens.

## Introduction

As a widespread contagious zoonotic disease affecting both animals and humans, and despite considerable efforts to control it, Brucellosis inflicts significant economic impacts on various regions worldwide while representing an important health risk to livestock [1]. *Brucella* spp. are facultative intracellular bacteria reported to have the capacity to evade or interfere host immune responses. They currently comprise 12 species, including *Brucella* (*B*.) *abortus*, which is one of the most important pathogenic species in humans [1, 2]. These bacteria affect a wide variety of host species in addition to humans and leading to serious debilitating disease with no short-course treatment regimens or licensed vaccines available for human patients [3]. Nevertheless, the number of new, human brucellosis cases remains unclear despite its significant recognition as a threat to both agricultural and public health sectors across the globe. At the same time, there is no sufficient empirical evidence regarding the incidence of the disease, and hence the World Health Organization (WHO) has withdrawn its priority Neglected Zoonotic Disease status [4]. Although a group of researchers in 2006 attempted to determine the incidence of human brucellosis and concluded the number of new infections to exceed half a million cases each year, the number was revealed to be not evidence based. Laine and colleagues [4] then conducted a study and suggested that with billions of vulnerable people in areas, where in many cases the existence of this infection was unknown, hundreds of millions could possibly be exposed to *Brucella* and therefore millions of people would likely contract the disease every year. This led to their recommendation that the Neglected Zoonotic Disease priority status for brucellosis be restored.

Brucella has a host cell tropism mostly to the monocyte/macrophage lineage but can also survive and replicate in nonphagocytic cells, such as fibroblasts and epithelial cells, and as a successful infectious agent requires four steps, including adherence, invasion, establishment and dissemination within the host [5-7]. Once inside the macrophage, the bacteria can survive, replicate, and control the fusion of the phagosome-lysosome complex to subsequently circulate to other cells of the host [1]. Among *Brucella* species, *B. abortus* is the strain most typically found in cattle populations. In South Korea, the numbers of human brucellosis cases and cattle outbreaks increased and decreased in the same pattern, as seen in the increased prevalence of brucellosis in Korean native cattle after the early 2000s, which was accompanied by an upward pattern of cases of human brucellosis [8, 9]. The prevalence of brucellosis in livestock is not decreasing, the available animal vaccines pose several drawbacks, and the treatment of the disease in humans consists of a harsh therapy using a combination of antibiotics for several weeks to months with occurrence of relapses and failures [1, 10, 11]. Such circumstances underscore the need to find alternative agents for treating brucellosis that are non-toxic, inexpensive, and effective.

Sirtuin 1 (SIRT1) is a member of the sirtuin family of NAD^+^-dependent deacetylases and the most studied among the seven members of the mammalian sirtuin family of which the primary function is deacetylation of post-translationally modified lysine residues [12]. Crotty Alexander *et al*. [13] reported that SIRT1 expression has little influence on macrophage and neutrophil antimicrobial functions utilizing both pharmacologic methods and a genetic knockout. Meanwhile, in a study by Opal *et al*. [12], SRT3025, a small molecule activator of SIRT1 known as SIRT1 activator compound (STAC), provided survival benefits such as promotion of bacterial clearance and reduced inflammatory cytokines from the lungs of *Streptococcus pneumoniae*-challenged mice which was absent in SIRT1 knockout. Resveratrol (RES) is the original STAC described, and is an antioxidant and polyphenol compound naturally found in peanuts, pistachio nuts, red grape seeds and skins, red wine and others, with a close structural similarity to that of synthetic estrogen diethylstilbestrol [12, 14]. This polyphenol has been reported for its chemopreventive activity possibly mediated directly via inhibiting cyclooxygenase-2 (COX-2) [15]. Gagnaire *et al*. [16] proposed the use of COX-2 inhibitors as novel strategies in controlling brucellosis. Piceatannol (PIC), on the other hand, is a natural analog of RES and a polyphenolic stilbene phytochemical present in grapes, mulberry leaves/fruits, nuts, passion fruits and berries, and is known to exhibit cardioprotective, antitumor, and antiaging effects [17]. Wang *et al*. [18] screened and identified a total of 19 SIRT1 activators from *Panax ginseng*, including ginsenoside Rg3. We previously reported the beneficial effect of Rg3 against *B. abortus* 544 infection in RAW264.7 cell line [19]. Here, we investigated the potential effects of RES, PIC and Rg3 against *B. abortus* 544 infection using RAW264.7 cells and a murine model with ICR mice.

## Materials and Methods

### Materials

RES (5-[2-(4-hydroxyphenyl)vinyl]benzene-1,3-diol, molecular weight, MW: 228.25 g/mol), PIC (4-[2-(3,5-dihydroxyphenyl)vinyl]benzene-1,2-diol, MW: 244.24 g/mol), 1% penicillin-streptomycin (10,000 U penicillin and 10 mg streptomycin/ml), streptomycin solution (1 mg/ml), 3-(4,5-dimethylthiazol-2-yl)-2,5-diphenyltetrazolium bromide (MTT), ethanol and dimethyl sulfoxide (DMSO), and ginsenoside Rg3 (MW: 785.01 g/mol) were obtained from Ambo Institute (Korea). RPMI 1640 was from Life Technologies Corporation (USA); fetal bovine serum (FBS) from Thermo Fisher Scientific (USA); BD cytometric bead array (CBA) mouse inflammation kit was purchased from BD Biosciences (USA); agar was from Yakuri Pure Chemicals Co., Ltd. (Japan), and *Brucella* broth came from Becton Dickinson (USA). *B. abortus* 544 biovar 1 (ATCC 23448) was kindly provided by the Laboratory of Bacteriology Division in Animal and Plant Quarantine Agency in Korea and was cultivated in 2%agar for at least 3 days at 37°C and cultured in broth for 2 days with shaking (180 rpm) prior to all infection assays. *Salmonella* Typhimurium (ATCC 14028) was cultivated in Luria Bertani (LB) agar or broth. Serial dilution using PBS was plated onto appropriate agar to determine the number of colony forming units (CFUs). Procedures on bacteria were done under Biosafety Level 3. RAW264.7 cells (TIB-71, USA) were maintained in RPMI 1640 supplemented with 10% heat-inactivated FBS and 1% penicillin-streptomycin incubated in a 5% CO_2_ atmosphere at 37°C. The cells were cultured in fresh medium without antibiotics prior to all infection assays. Seven-week-old pathogen-free female ICR mice were purchased from Samtako Bio Co. Ltd. (Korea), housed in metabolic cages (12 h light/12 h dark cycle), and acclimated for 7 days with *ad libitum* access to food and water.

### Determination of Cell Viability

Viability of cells was detected using MTT assay. RAW264.7 cells were cultured overnight in a 96-well plate at a concentration of 1 × 10^5^ cells per well and then incubated with different concentrations of RES (0, 5, 10, 20, 30, 40, 60 and 80 μM), PIC (0, 1, 3 and 10 μM) and Rg3 (0, 130, 1300, 13000 μM) for 48 h. At the end of the incubation period, the cells were washed and the medium was changed to RPMI 1640 with MTT reagent (5 mg/ml) and incubated for 2 h. The medium was removed and 150 μl of DMSO was added to each well and further incubated for 15 min. The absorbance was measured at 540 nm and the percent viability was computed in comparison to the vehicle (0.1% ethanol in fresh medium).

### Adhesion, Internalization, and Intracellular Killing Assay

The treatment control groups for RES, PIC and Rg3 were 0.1% ethanol, 0.1% DMSO, and PBS, respectively. RAW264.7 cells were prepared following the protocol performed for the determination of cell viability. For adhesion and internalization assays, cells were pre-incubated with RES (1, 10 and 20 μM), PIC (0.5, 1 and 10 μM), Rg3 (13, 64 and 130 μM) for at least 4 h. Cells were then washed and the medium was changed prior to infection with *B. abortus* at multiplicity of infection (MOI) of 50. The plate was centrifuged in a sealed carrier at 200 ×*g* for 5 min and further incubated at indicated times. For adhesion assay, infected cells were washed at 0.5 h post-infection time to remove unassociated bacteria as previously described [20]. The cells were then lysed using 100 μl distilled water, pipette mixed, and 10 μl was diluted in PBS. Fifty microliters was plated onto *Brucella* agar to determine CFU. For internalization assay, infected cells were washed at least two times using PBS at 0, 0.5 and 1 h post-infection and incubated in fresh medium containing 10% FBS and 100 μg/ml gentamicin for 0.5 h. Washing, lysis, dilution and plating were the same as for the adhesion assay. For intracellular growth assay, RAW264.7 cells were first infected with *B. abortus* for 1 h. The cells were washed at least two times using PBS and the culture medium was changed to fresh medium containing 10% FBS, 100 μg/ml gentamicin and RES, PIC, Rg3 or PBS and incubated for 0.5 min. The culture medium was changed with a lower concentration of gentamicin (30 μg/ml) and further incubated for a total of 2, 24, and 48 h. Washing, lysis, dilution and plating were the same as for the adhesion assay. Adhesion, internalization and intracellular growth efficiencies for *Salmonella* Typhimurium were also determined using the highest concentration of RES (20 μM), PIC (10 μM) and Rg3 (100 μg/ml).

### *B. abortus* Infection In Vivo

Procedures on animals complied with the established federal guidelines and institutional policies of the Animal Ethical Committee of Chonbuk National University (Authorization No. CBNU-2018-00374). Mice were randomly distributed into six groups of six animals each: RES, PIC and Rg3 with their respective control groups (0.1%ethanol, 0.1% DMSO and PBS) for three weeks and were monitored for any clinical symptoms. Animals received oral treatment of a total volume of 100 μl via feeding needle of RES (20 μM), PIC (10 μM), Rg3 (130 μM) and their respective vehicle. After one week, blood was collected via tail vein and mice were infected the next day with *B. abortus* at a concentration of 2 × 10^4^ CFU diluted in PBS in a total volume of 100 μl via intraperitoneal route. Blood was collected again at 7 and 14 days post-infection (dpi), and the mice were sacrificed at 15 dpi. Spleen and liver were immediately collected since these are the primary organs affected by *Brucella*. Each organ was weighed aseptically and a 0.05 g part was collected, homogenized in PBS, and serially diluted and plated onto *Brucella* agar to determine CFU/g of organ. Serum was collected from the blood samples to analyze cytokine production during the entire treatment and infection period.

### Cytokine Analysis

Fifty microliters of serum samples was processed to quantify the level of IL-12p70, TNF, IFN-γ, MCP-1, IL-10 and IL-6 using a CBA mouse inflammation kit following the manufacturer’s instructions. Data were acquired on a FACSCalibur flow cytometer (BD Biosciences, USA), analyzed using BD CellQuest™ software, and then finally sent to BD company to measure the cytokine level.

### Statistical Analyses

In vitro experiments were performed at least three times with at least two replicates while in vivo experiments involved six animals per group. Data are represented as the means ± SD of independently performed experiments. All data analyses, computations and graphs were performed using GraphPad InStat software version 3 and GraphPad Prism 5.03 (GraphPad Software, Inc., USA). Comparisons between groups were assessed by Student’s *t*-test and a value of *p* < 0.05 was considered significant.

## Results

### Effects of SIRT1 Activators on the Proliferation of *B. abortus* in RAW264.7 Cells

**Fig. 1:**
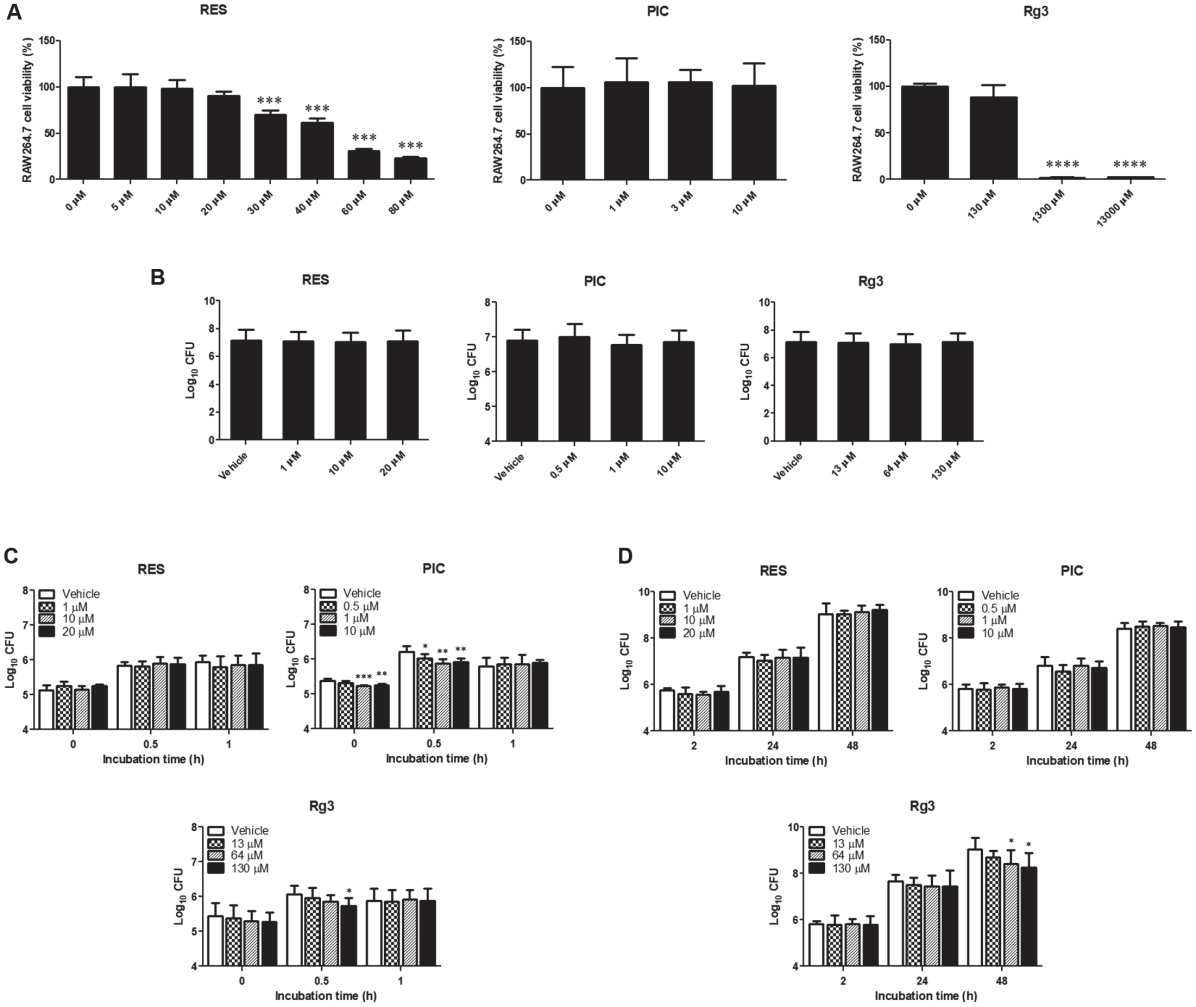
Effect of SIRT1 activators in *Brucella*-infected RAW264.7 macrophages. Cells were incubated with different concentrations of RES (0, 5, 10, 20, 30, 40, 60 and 80 μM), PIC (0, 1, 3 and 10 μM) and Rg3 (0, 130, 1300, 13000 μM) for 48 h (**A**). Cells were pre-treated with RES (1, 10 and 20 μM), PIC (0.5, 1 and 10 μM) or Rg3 (13, 64 and 130 μM) for at least 4 h prior to infection and *Brucella* adherence (**B**) and internalization (**C**) efficiencies at indicated times were determined. Cells were also infected with *Brucella* prior to incubation with RES, PIC or Rg3 and the intracellular survival of *Brucella* was calculated (**D**). Data represent the mean ± SD. Statistically significant differences relative to the control vehicle are indicated by asterisks (*, *p* < 0.05; **, *p* < 0.01; ***, *p* < 0.001; ****, *p* < 0.0001).

First, the highest non-cytotoxic concentration of RES was determined. Overnight culture of RAW264.7 cells was treated with different concentrations of RES and the results showed that 20 μM was the highest concentration that did not affect the viability of the cells after 48 h of incubation (Fig. 1A). The highest concentrations used for PIC and Rg3 were 10 μM and 130 μM, respectively, in reference to previous reports [19, 21] as well as in the present study (Fig. 1A). These concentrations were used in the succeeding experiments in both in vitro and in vivo tests. The efficiency of *Brucella* to adhere in RAW264.7 cells was determined and the results showed that none of the treatment groups affected the ability of the bacteria to attach to these cells (Fig. 1B). Next, we examined the efficiency of the bacteria to enter the cells and found that RES treatment did not alter the number of internalized bacteria at any concentrations tested at all time points (Fig. 1C). Interestingly, a reduced internalization efficiency was observed in cells treated with PIC and Rg3. At 0 h post-infection, a lower number of internalized bacteria was observed in PIC-treated cells at 1 and 10 μM concentrations, and all of the concentrations tested were observed to cause reduction in the number at 0.5 h post-infection (Fig. 1C). In Rg3-treated cells, only the highest concentration at 0.5 h post-infection was observed to inhibit the number of internalized bacteria into these cells (Fig. 1C). No effect was observed in all of the treatments at 1 h post-infection. On the other hand, intracellular growth efficiency was observed to change only in Rg3-treated cells, which was observed at 48 h post-incubation in a dose-dependent manner (Fig. 1D). Overall, the results suggest that the SIRT1 activators used in the present study had no effect on the attachment of *Brucella* into RAW264.7 cells but had different mechanisms of action in the aspects of the phagocytic and intracellular pathways.

### Effects of SIRT1 Activators on the Proliferation of Other Intracellular Pathogens

**Fig. 2:**
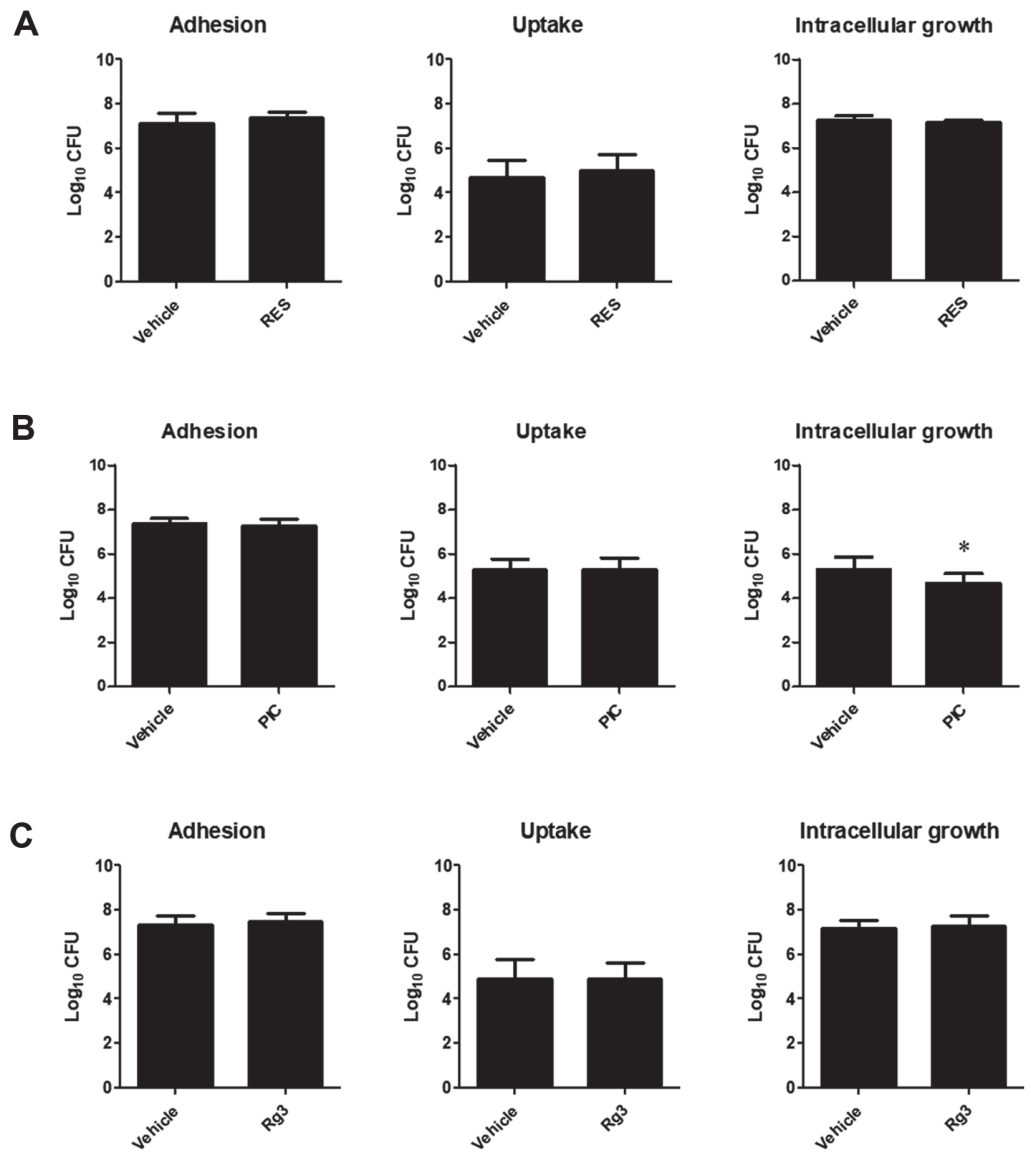
Effect of SIRT1 activators in *Salmonella*-infected RAW264.7 macrophages. Cells were pre-treated with RES (20 μM), PIC (10 μM) or Rg3 (130 μM) for at least 4 h prior to infection with *Salmonella*. Adherence, internalization and intracellular survival efficiencies of *Salmonella* were determined in cells incubated with RES (**A**), PIC (**B**) or Rg3 (**C**). Data represent the mean ± SD. Statistically significant differences relative to the control vehicle are indicated by asterisks (*, *p* < 0.05).

We also determined the effect of RES, PIC and Rg3 using their highest non-cytotoxic concentration and compared the number of CFUs with that of their corresponding control groups on the adhesion, uptake, and intracellular survival of another intracellular pathogen using *Salmonella* Typhimurium. Results showed that RES treatment had no effect (Fig. 2A) while PIC treatment had significantly attenuated the intracellular growth of *Salmonella* at 24 h post-incubation (Fig. 2B). Rg3 treatment did not alter the adhesion, internalization, and intracellular survival efficiencies of *Salmonella* in RAW264.7 cells (Fig. 2C).

### Effects of SIRT1 Activators on the Proliferation of *B. abortus* in Mice

**Fig. 3:**
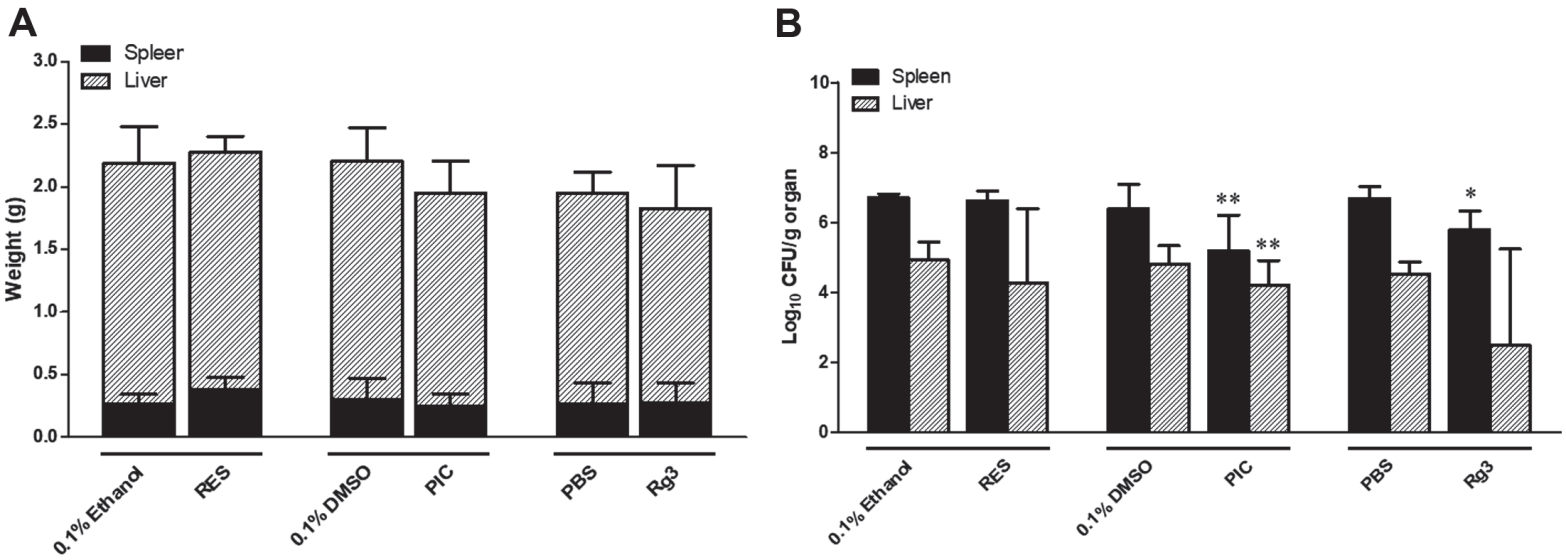
Effect of SIRT1 activators in the susceptibility of mice against *Brucella* infection. ICR mice were randomly grouped and each group was orally treated with RES (20 μM), PIC (10 μM) or Rg3 (130 μM) for one week with their corresponding control groups. Animals were intraperitoneally infected with *Brucella* at a concentration of 2 × 10^4^ CFU and the oral treatment was continued for two more weeks. Mice were sacrificed at 15 dpi and the spleens and livers were individually and aseptically removed. The organs were weighed (**A**) and the log10 CFU/g organ was calculated (**B**). Data represent the mean ± SD. Statistically significant differences relative to the control vehicle are indicated by asterisks (*, *p* < 0.05; **, *p* < 0.01).

To evaluate whether RES, PIC and Rg3 are able to inhibit *B. abortus* infection in mice, oral treatment was performed for 7 days prior to infection and continued until 14 dpi. The average weight of spleens and livers collected from mice treated with RES, PIC or Rg3 was not different from their corresponding control groups (Fig. 3A). Significant reduction in the proliferation of *Brucella* in spleens and livers was observed in mice that received PIC treatment, while only an attenuated number of CFUs in the spleens was observed in Rg3-treated mice (Fig. 3B). No changes were observed in mice that were orally given RES although a lower number of CFUs was observed in the livers (Fig. 3B).

### Effects of SIRT1 Activators on the Serum Level of Different Cytokines Involved in *B. abortus* Infection in Mice

**Fig. 4:**
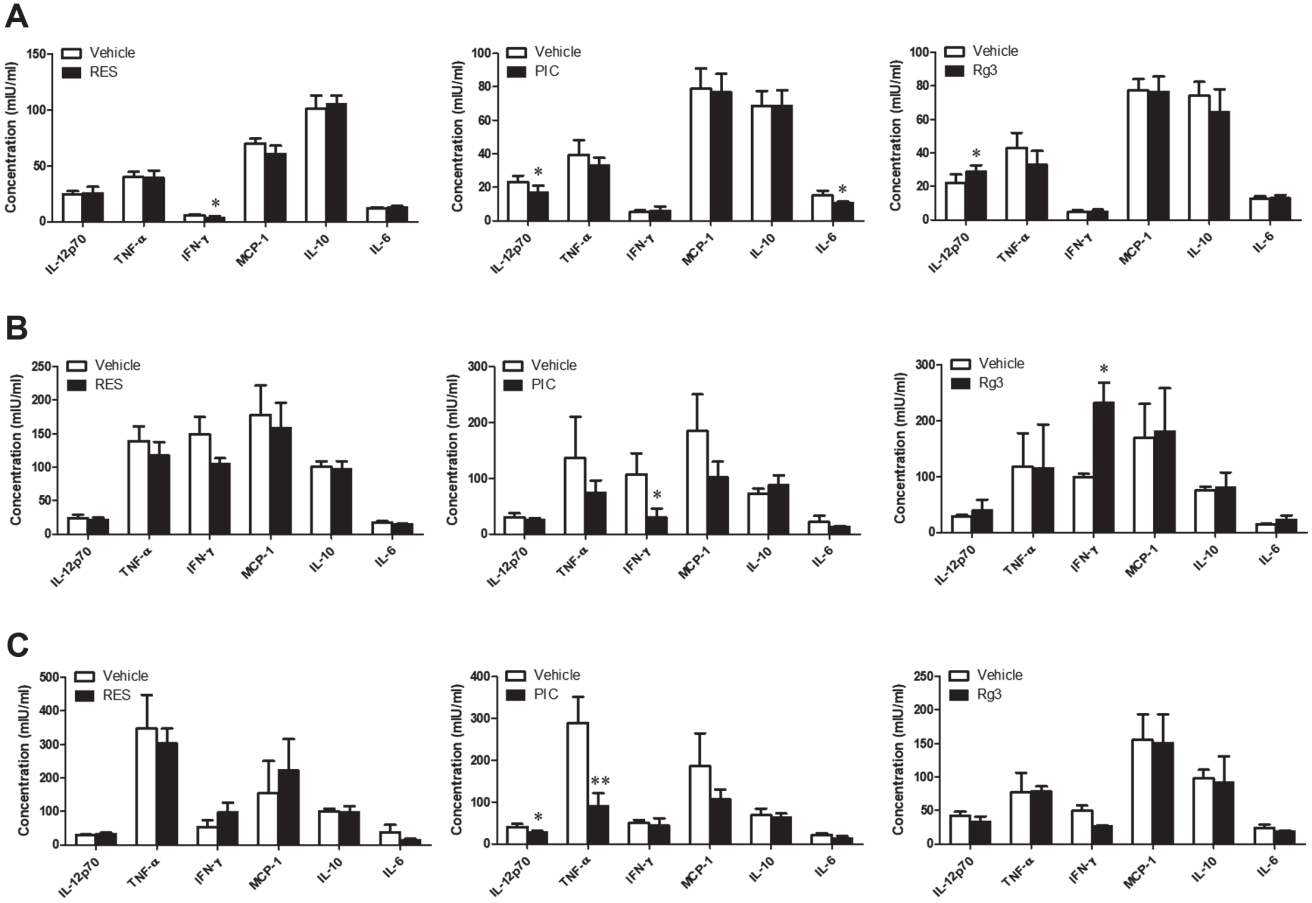
Effect of SIRT1 activators in the in the serum production of cytokines in mice. The cytokine levels were determined in ICR mice treated with RES (20 μM), PIC (10 μM) or Rg3 (130 μM) at 7 days post-treatment (**A**), 7 dpi (**B**) and 14 dpi (**C**). Data represent the mean ± SD. Statistically significant differences relative to the control vehicle are indicated by asterisks (*, *p* < 0.05; **, *p* < 0.01).

At 7 days post-treatment, blood was analyzed for serum levels of the different cytokines involved during *Brucella* infection to evaluate immune regulation during SIRT1 activator treatment in mice. Reduced serum level of IFN-γ was observed in mice that were orally given with RES, lower IL-12 and IL-6 serum levels were observed in mice that received PIC treatment while increased IL-12 was observed in Rg3-treated mice (Fig. 4A). These findings suggest the anti-inflammatory effects of RES and PIC, while promotion of inflammation could be attributed to Rg3 treatment. At 7 dpi, no changes in the serum cytokine levels were observed in RES-treated mice; lower serum level of IFN-γ was observed in PIC-treated mice, but this cytokine was observed to increase in mice that received Rg3 (Fig. 4B). Lastly, at 14 dpi, increased level of TNF-α was observed in RES-treated mice while attenuated serum levels of IL-12 and TNF-α were observed in PIC-treated mice (Fig. 4C). No significant changes in the cytokine level were observed in Rg3-treated mice (Fig. 4C). These results suggest different mechanism of action between the different SIRT1 activators in the context of immunoregulation in animal host.

## Discussion

Brucella is considered a highly infectious agent for its ability to establish infection in humans via the aerogenous route with an estimated dose of only 10-100 organisms [22]. Adherence, invasion, establishment, and dissemination within the host are the required steps for any pathogenic intracellular bacterium to be a successful infectious agent such as *Brucella* [23]. *Brucella* replicates and establishes infection in macrophages/monocytes, which are its primary target cells [24]. Here, we demonstrated that SIRT1 activators RES, PIC and Rg3 did not alter the adherence efficiency of either *Brucella* or *Salmonella* in a murine macrophage cell line RAW264.7, but act in the favor of the host cell in reducing internalized bacteria, particularly in PIC and Rg3 treatment. Chen *et al*. [25] reported that SIRT1 could directly interact with c-Fos and c-Jun basic leucine zipper domains that suppress AP-1 transcriptional activity and subsequent reduction of COX-2 expression. Hence, SIRT1 overexpression in macrophages reduced prostaglandin E2 which is an inhibitor of the phagocytic activity and bacterial killing. However, this is in contrast to the inhibitory effect observed in the action of PIC and Rg3 that resulted in the attenuation of the number of bacteria entering the macrophages. Interestingly, possible intracellular killing due to a lower number of bacteria recovered from PIC- and Rg3-incubated post-infected RAW264.7 cells against *Salmonella* and *Brucella*, respectively, could be attributed to its action on COX-2; however, this needs to be examined carefully in future studies in the context of intracellular pathogen infection. Nevertheless, these findings suggest a different mechanism of action for the control of internalized bacteria. In contrast to a report that SIRT1 expression has little influence on macrophage and neutrophil antimicrobial functions [13], the results in the present study suggest the possibility of SIRT1/COX-2-dependent microbial control within host cells.

SIRT1 has been associated with prevention of viral diseases, but its role in chronic bacterial infections has not been widely studied. However, its contribution against *Mycobacterium* (*M.*) *bovis* infection has already been investigated. Cheng *et al*. [26] reported that SIRT1-expressing cells were mainly macrophages and its downregulation is mediated by *M. bovis*. Furthermore, lower SIRT1 expression is connected with *M. bovis* infection as well as the pathogenesis of tuberculosis, indicating SIRT1 activators as a potential and effective host-directed therapeutic approach against tuberculosis. *Brucella*, when not treated properly, can lead to chronic infection that leads to severe health problems [27]; however, while the use of RES in the present study did not alter *Brucella* infection in either in vitro or in vivo experiments, other reported SIRT1 activators including PIC and Rg3 showed decreased susceptibility to *Brucella* infection. In a study by Nijampatnam *et al*. [28], a significant reduction in *Streptococcus mutans* in the gut of flies after 7 days of infection and a reduction in dental caries in a rat model accompanied by reduced but insignificant bacterial colonization was observed with PIC treatment suggesting that PIC inhibits *S. mutans* colonization or inhibits virulence factors in vivo. Rg3, on the other hand, had protective effect on *Escherichia coli* lipopolysaccharide-induced stress in chicks showing ameliorated growth inhibition and fever, and decreased production of stress-related hormones, possibly mediated by regulating inflammatory response and oxidative damage [29].

SIRT1 has been indicated to suppress innate inflammatory responses, but other studies reported otherwise [13]. Here, in vivo treatment with SIRT1 activators, particularly RES and PIC, showed reduced serum levels of IL-12, IFN-γ, TNF-α, and IL-6, which suggest inactivation of pro-inflammatory response during *B. abortus* infection. However, only PIC treatment is accompanied by augmented *Brucella* resistance in mice. Several studies on SIRT1 silencing or inactivation showed promotion of pro-inflammatory cytokine production, such as SIRT1 deletion or inactivation treatment in murine dendritic cells (DCs) that increased production of IL-12, which is a heterodimeric pro-inflammatory cytokine that induces IFN-γ production [25, 30]. IL-6, on the other hand, has also been demonstrated to be required for IFN-γ in addition to TNF-α [31]. NF-ĸB p65 hyperacetylation in SIRT1-deficient macrophages results in increased level of pro-inflammatory cytokines including TNF-α [32]. Therefore, suppression of inflammatory response could be due to inhibition of NF-ĸB activity via deacetylation of NF-ĸB p65. The reduction in the susceptibility to *Brucella* infection by PIC treatment despite attenuated pro-inflammatory cytokine production could be explained by an IFN-γ/IL-10 ratio of less than 1, hence suggesting a favorable Th2 immune response that is known to be important in the eradication of extracellular parasites and bacterial infection. Kim *et al*. [33] reported the transient IFN-γ production that promotes *B. abortus*-induced abortion using ICR mice. This then suggests the potential use of RES or PIC as an effective approach against extracellular parasites and prevention of abortion in animal brucellosis. However, Rg3 treatment in mice displayed induced serum levels of IL-12 and IFN-γ, suggesting a different mechanism of action from that of PIC treatment, but with a similar, corresponding favorable outcome against *Brucella* infection. This suggests also the potential use of Rg3 as a vaccine adjuvant. IL-12 is known to contribute to *B. abortus* resistance mainly via IFN-γ-dependent pathway, while IFN-γ has also been suggested to enhance resistance via activation of macrophages for brucellicidal activity [34, 35]. The differences in the in vitro and in vivo results could be due to the controlled environment using cell lines while in vivo tests include the whole living organism that could involve several factors in the action of the different activators used in the present study. Furthermore, our findings suggested the variation and specificity of the actions of the different SIRT1 activators. Nevertheless, further investigations are necessary for a complete understanding of the mechanisms involved in the control of *Brucella* infection using SIRT1 activators. Taken together, the use of SIRT1 activators individually or in combination could be a potential, effective alternative approach in the control of intracellular infection such as *Brucella* and *Salmonella*.

## Figures and Tables

**Fig. 5:**
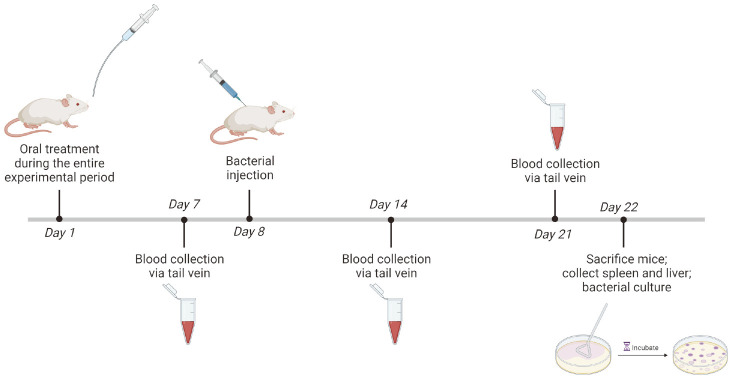
Brucella infection in mice. Animals received oral treatment via feeding needle of RES (20 μM), PIC (10 μM), Rg3 (130 μM) and their respective vehicle. Blood was collected via tail vein at 7 days post-treatment. Mice were infected the next day with *B. abortus* via intraperitoneal route. Blood was collected again at 7 and 14 dpi. The animals were sacrificed at 15 dpi and the spleen and liver were aseptically collected. A 0.05-gram section of the organ was collected, homogenized in PBS and serially diluted and plated onto *Brucella* agar to determine CFU at 3 days post-incubation of plates.
